# Crystal structure and Hirshfeld surface analysis of 2-picolyllithium·3thf

**DOI:** 10.1107/S2056989023010873

**Published:** 2024-01-01

**Authors:** Tristan Mairath, Annika Schmidt, Carsten Strohmann

**Affiliations:** a TU Dortmund University, Fakultät für Chemie und chemische Biologie, Anorganische Chemie, Otto-Hahn-Strasse 6, 44227 Dortmund, Germany; University of Aberdeen, United Kingdom

**Keywords:** crystal structure, 2-picolyllithium, 2-methyl­pyridyl­lithium, Hirshfeld surface analysis

## Abstract

In the title compound, (2-methyl­idene-1,2-di­hydro­pyridinium-κ*N*)tris­(tetra­hydro­furan-κ*O*)lithium, [Li(C_6_H_6_N)(C_4_H_8_O)_3_], the lithium ion adopts a distorted LiNO_3_ tetra­hedral coordination geometry and the 2-picolyl anion adopts its enamido form with the lithium ion lying close to the plane of the pyridine ring. A methyl­ene group of one of the thf ligands is disordered over two orientations. In the crystal, a weak C—H⋯O inter­action generates inversion dimers. A Hirshfeld surface analysis shows that H⋯H contacts dominate the packing (86%) followed by O⋯H/H⋯O and C⋯H/H⋯C contacts, which contribute 3% and 10.4%, respectively.

## Abstract 2

In the title compound, the lithium ion adopts a distorted LiNO_3_ tetra­hedral coordination geometry and the 2-picolyl anion adopts its enamido form with the lithium ion lying close to the plane of the pyridine ring. In the crystal, a weak C—H⋯O inter­action generates inversion dimers. A Hirshfeld surface analysis shows that H⋯H contacts dominate the packing (86%) followed by O⋯H/H⋯O and C⋯H/H⋯C contacts, which contribute 3% and 10.4%, respectively.

## 1. Chemical context

**Figure 1:**
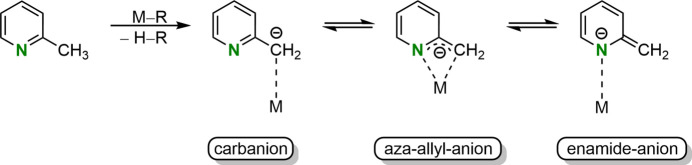
Transformation of 2-picoline into its carbanion, aza-allyl anion and enaminde anion forms.

Among the various synthetic approaches for the introduction of 2-picoline (C_6_H_7_N) into a wide range of chemical products, the route *via* a metallated inter­mediate (*i.e*., the 2-picolyl anion, C_6_H_6_N^−^) followed by trapping with an electrophile has proven to be particularly attractive due to the large number of possible electrophilic compounds. The formation of these metal-containing inter­mediates usually takes place by reaction with organometallic bases such as lithium organyles (Gessner *et al.*, 2009 ▸), resulting in deprotonation of the picoline and consequent anion formation (Beumel Jr *et al.*, 1974 ▸). Due to resonance-stabilizing effects, there are different possibilities to stabilize the negative charge formed at the 2-picoline moiety. In addition to the delocalization of charge across the aromatic ring, further anionic motifs in the sense of a carbanion, an aza-allyl anion, or an enamide anion are possible: see Fig. 1 ▸.

**Figure 2:**
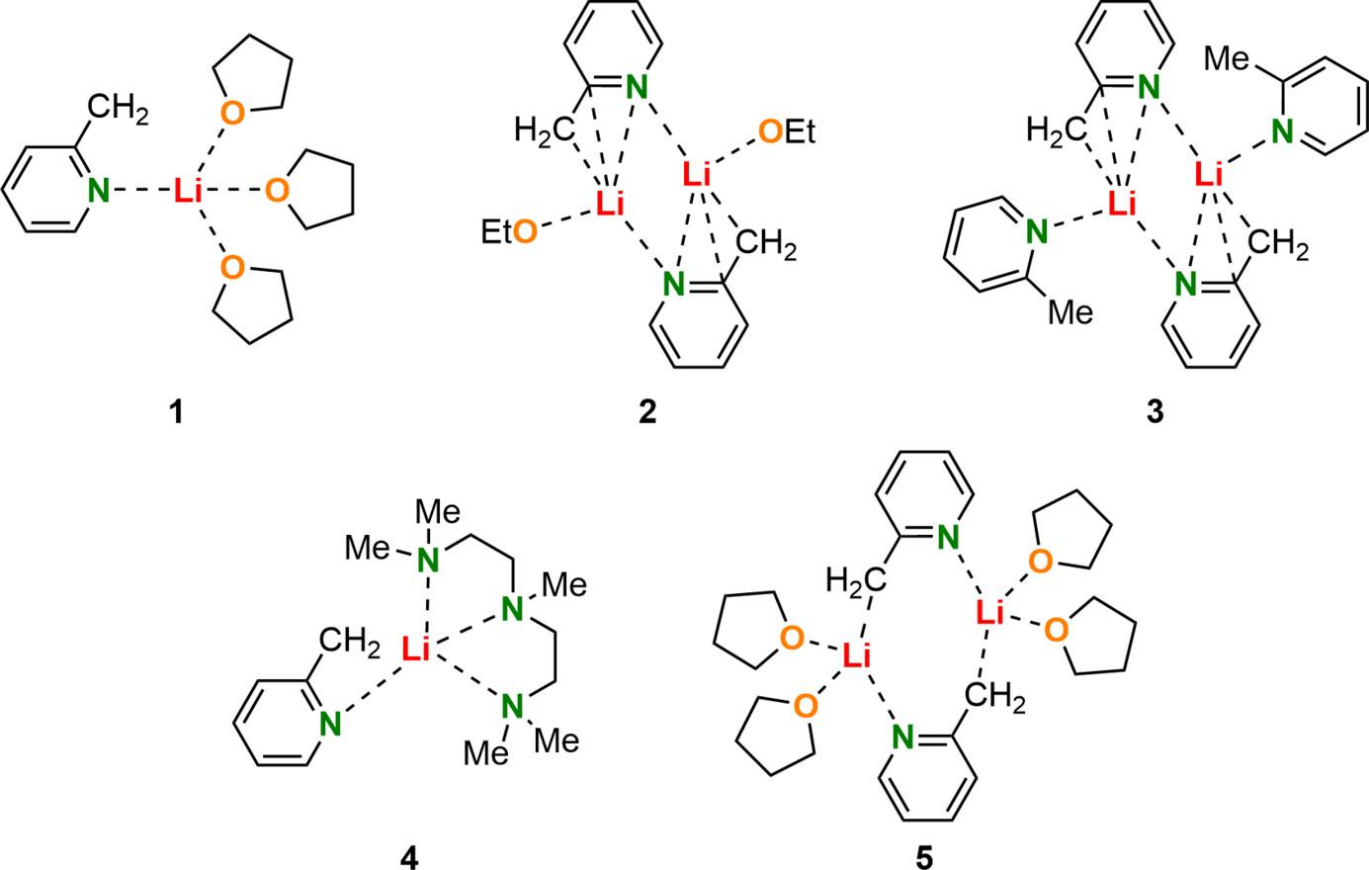
Structures of the title compound (2-PicLi·3thf) (**1**), (2-PicLi·OEt_2_)_2_ (**2**), (2-PicLi·PicH)_2_ (**3**), enamido (2-PicLi·pmdta) (**4**) and dimeric carbanionic [2-PicLi·(thf)_2_]_2_ (**5**).

Charge-density studies by Ott *et al.* (2009 ▸) confirmed the existence of the aza-allyl carbanionic 2-picolyl motif by solid-state analysis of two dimeric 2-picolyllithium structures (2-PicLi·OEt_2_)_2_ (**2**) and (2-PicLi·PicH)_2_ (**3**). Both structures are defined by two different lithium–anion inter­actions within one complex (Fig. 2 ▸). On the one hand there is an Li—N bond such that the metal ion lies almost coplanar to the aromatic pyridyl ring and on the other hand an *η* ^3^-aza-allylic contact can be identified. While NBO analysis determined partial negative charges at the nitro­gen atom (–0.78 e) and formed carbanion (–0.69 e), which indicates aza-allylic character, bond-path analysis could only identify a bond path between the lithium and nitro­gen atoms. In conclusion, the Li—N inter­action was described as more dominant and the Li–carbanion contact as an auxiliary inter­action (Ott *et al.*, 2009 ▸).

The group of Mulvey (Kennedy *et al.*, 2014 ▸) followed up on these studies and reported the monomeric solid-state structure (2-PicLi·pmdta) (**4**) (pmdta = *N*,*N*,*N*′,*N*′′,*N*′′-penta­methyl­diethylenetri­amine, C_9_H_23_N_3_). In contrast to the dimeric aza-allyl motif **2** of Stalke *et al.*, Mulvey and co-workers identified the monomeric structure **4** as an enamido motif due to the sole Li—N inter­action (Fig. 1 ▸). Saturation of the lithium coordination sphere is accomplished by the chelating pmdta ligand. To characterize the described solid-state structures, the location of the lithium cations relative to the aromatic pyridyl ring serves as an important tool. Therefore aza-allylic structures like **2** or **3** were defined by *sp* ^2^-hybridized nitro­gen atoms and C_*para*_—N—Li bond angles of about 180°, representing an almost planar arrangement. The enamido motif shows a divergent C_*para*_—N—Li angle of about 146° indicating *sp* ^3^-hybridization of the nitro­gen center (Kennedy *et al.*, 2014 ▸). Due to the usage of different solvents, a follow-up dimeric structure [2-PicLi·(thf)_2_]_2_ (**5**) could be obtained by Brouillet *et al.* (2020 ▸) (Fig. 2 ▸). Unlike the previous dimeric structure **2** of Stalke *et al.*, NBO calculations determined negative charges at N (–0.68 e), O (–0.65 e) and C2 (–0.80 e) suggesting a carbanionic structural motif. Thus, all three possible structural motifs have been detected and characterized in the solid state (Brouillet *et al.*, 2020 ▸).

In this work, using an excess amount of the tetra­hydro­furan (thf) ligand, a related structure to [2-PicLi·(thf)_2_]_2_ (**5**) by Mulvey *et al.* was obtained in the form of the title li­thia­ted monomeric 2-picoline saturated by three thf mol­ecules [2-PicLi·(thf)_3_] (**1**) (Fig. 1 ▸). Inter­estingly, this monomeric structure shows an inconsistent C ***_para_*** ⋯N—Li angle of 179.9° regarding to former enamido motifs, indicating an *sp* ^2^-hybridized nitro­gen in contrast to usual *sp* ^3^-hybridization. 
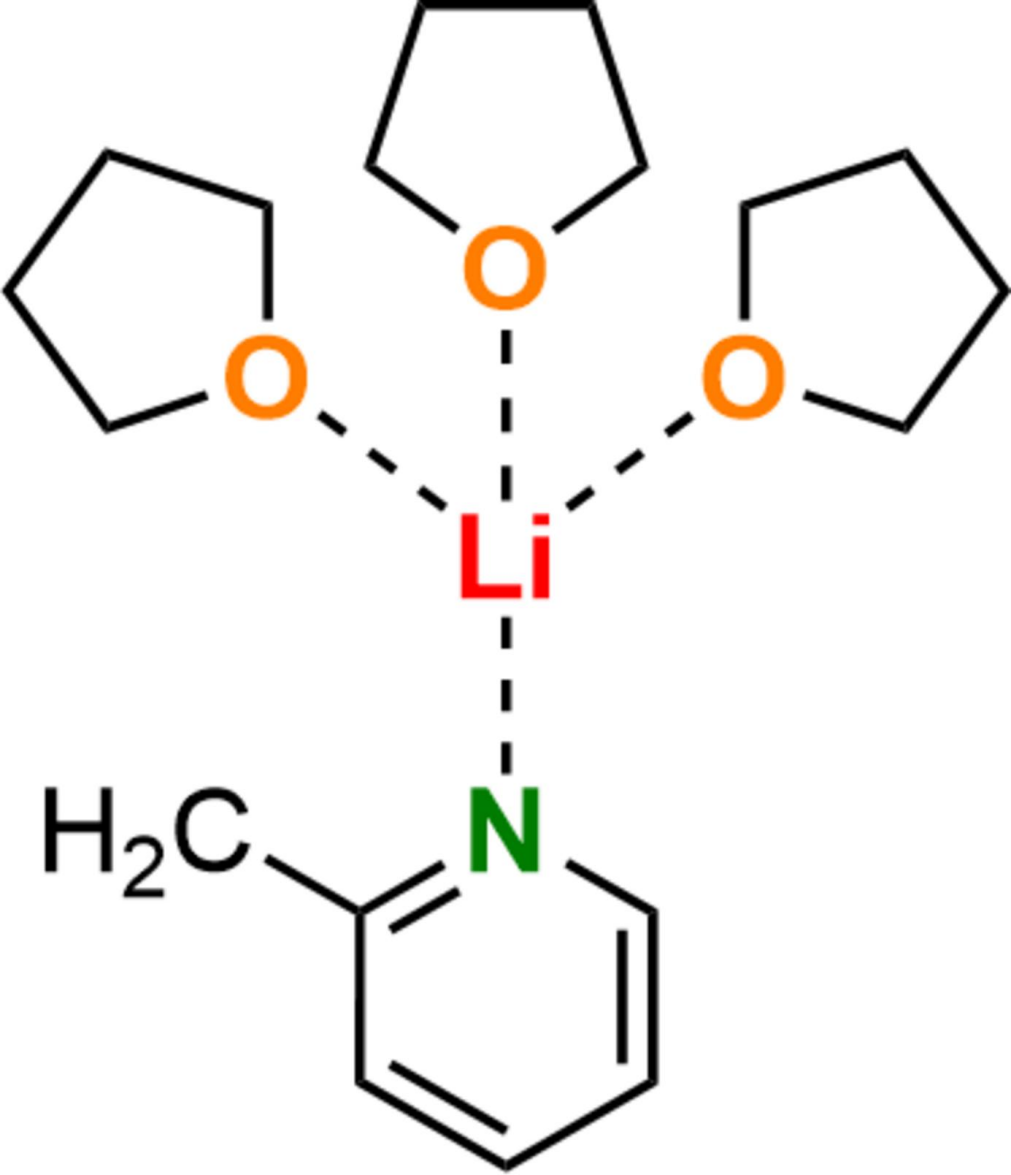


## 2. Structural commentary

**Figure 3:**
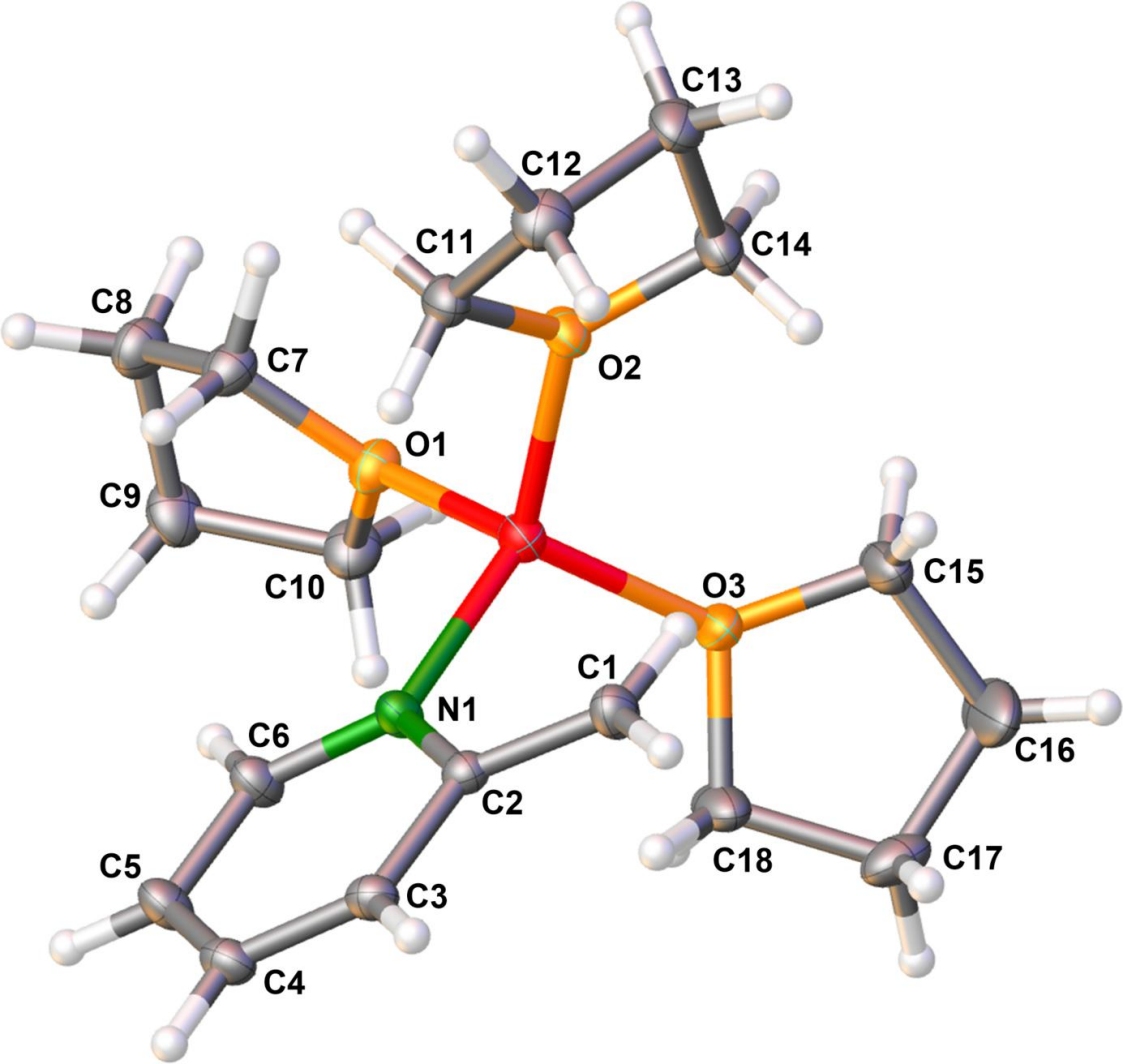
The mol­ecular structure of compound **1** with displacement ellipsoids drawn at the 50% probability level. Only the major disorder component is shown.

Fig. 3 ▸ shows the mol­ecular structure of **1** and selected bond lengths and angles are given in Table 1 ▸. The solid-state structure consists of a li­thia­ted 2-picoline unit forming an enamido motif. The lithium cation is coordinated by the N atom of 2-picoline as well as by three thf mol­ecules. The O—Li1—N1 angles of 106.33 (7), 115.29 (7) and 111.51 (7)° indicate a slightly distorted tetra­hedral coordination, probably due to packing effects (see *Supra­molecular features*). Li­thia­tion led to deprotonation of the methyl substituent resulting in *sp* ^2^-hybridization of the C1-carbon atom, which is recognizable due to shortening of the C1—C2 bond and the changing sum of bond angles to 360° at the carbanionic center, compared to the solid-state structure of 2-picoline (Bond & Davies, 2001 ▸). With a length of 1.3804 (10) Å, the C1—C2 bond is significant shorter than typical C*sp* ^2^—C*sp* ^2^ single bonds (1.466 Å) but too long for C*sp* ^2^—C*sp* ^2^ double bonds (1.335 Å; Rademacher, 1987 ▸). This is caused by stabilization of the negative charge by the aromatic ring. Due to the shortened C1—C2 bond, the overall bonding situation in the aromatic ring is changed as well, displayed by extended C2—C3 [1.4548 (19) Å], C4—C5 [1.4196 (12) Å] bonds and shortened C3—C4 [1.3664 (11) Å] and C5—C6 [1.3855 (11) Å] bonds. While the N1—C2 bond length increased by about 0.06 Å, the N1—C6 bond length is comparable to the equivalent bond in the educt structure (Bond & Davies, 2001 ▸).

The coordination distance Li1—N1 is only slightly longer than in the related monomeric structure of li­thia­ted 2-picoline with pmdta, **4**. However, this can be explained by stronger coordinating thf ligands characterized by shorter Li—O distances [1.9493 (16) to 1.9698 (15) Å] compared to the nitro­gen coordination distance of pmdta [2.138 (7) to 2.147 (7) Å]. One thf ligand of **1** shows disorder of one of its methyl­ene groups over two adjacent positions in a 0.717 (5): 0.283 (5) ratio.

Another striking feature of the monomer **1** is the planar arrangement of the lithium cation relative to the aromatic ring. As indicated by the angle Li1—N1⋯C4 of 179.9°, the cation hardly deviates from the ring plane. Together with the angular sum of 360° around N1, an *sp* ^2^-hybridized nitro­gen atom can be assumed. According to this, the lithium cation should be coordinated by a dative bond based on the free electron pair of the nitro­gen. This is in strong contrast to the monomeric compound **4** observed by Mulvey *et al.* in which an Li1—N1—C4 angle of 145.9 (2)° was observed, which suggests *sp* ^3^-hybridization of the nitro­gen center and coordination of the lithium cation *via* a localized negative charge.

A greater similarity with **1** is shown by the dimeric carbanionic structure of li­thia­ted 2-picoline with thf, **5**. The dimer consists of a non-planar eight-membered (NCCLi)_2_ ring in the solid state. A planar arrangement of the lithium cation with the aromatic ring was observed and the authors describe a dative coordination of the cation *via* an *sp* ^2^-hybridized nitro­gen atom. However, the Li1—N1 coordination in **5** is described as a weaker inter­action, as in the case of the *sp* ^3^-hybridized nitro­gen atom in structure **4**. Therefore, the carbanionic CH_2_ substituent of **5** induces a stronger coordination to the lithium cation. In **1**, less carbanionic character of the CH_2_ substituent is detectable, due to delocalization of the charge to the aromatic ring. The significantly shortened C1—C2 bond and the angular sum at the C1 atom of 360° indicate *sp* ^2^ hybridization. This would be more comparable to the monomeric structure of Mulvey *et al.*

In summary, the here-presented structure **1** shows features of both structures **4** and **5**. While the *sp* ^2^ hybridization of the CH_2_ substituent is more similar to the monomeric structure **4**, the linear arrangement of Li1—N1⋯C4 and the resulting presumed *sp* ^2^ hybridization of the nitro­gen atom is more comparable to the dimeric structure **5**.

## 3. Supra­molecular features

**Figure 4:**
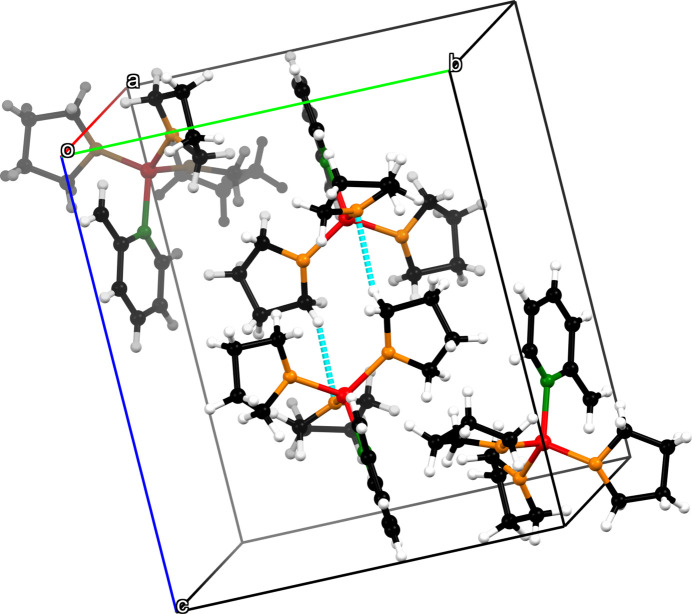
The crystal packing of compound **1**. C—H⋯O hydrogen bonds are shown as dashed blue lines.

**Figure 5:**
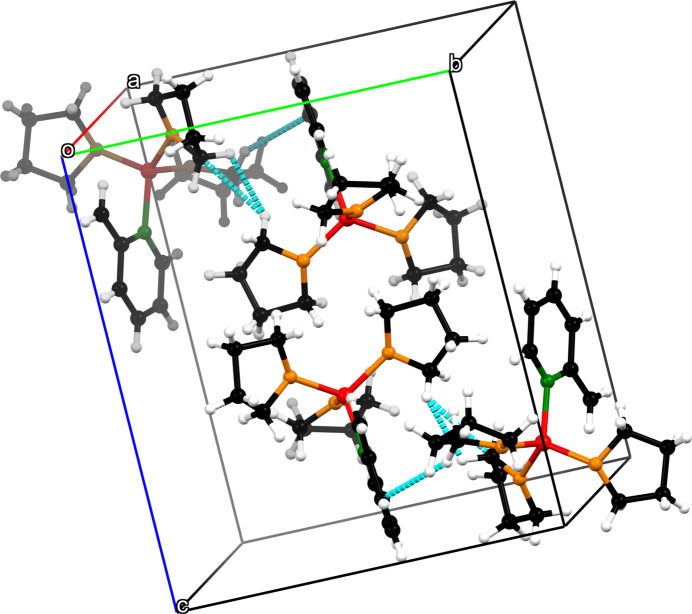
The crystal packing of compound **1**. C11^i^/H11*B* ^i^⋯H19*B* and H7*A* ^i^⋯C3 van der Waals inter­actions are shown as dashed blue lines.

An important supra­molecular structural element of compound **1** is the two close contacts between O1 and H15*B* across the inversion center (Fig. 4 ▸). With a coordination distance of O1^i^⋯C15 = 3.3695 (14) Å [symmetry code: (i) 1 − *x*, 1 − *y*, 1 − *z*], fairly long-range inter­actions are represented. Due to two inter­molecular C—H inter­actions (Table 2 ▸) between C11^i^/H11*B* ^i^ and H19*B* as well as H7*A* ^i^ and C3, further coordination points are given in the solid state (Fig. 5 ▸).

**Figure 6:**
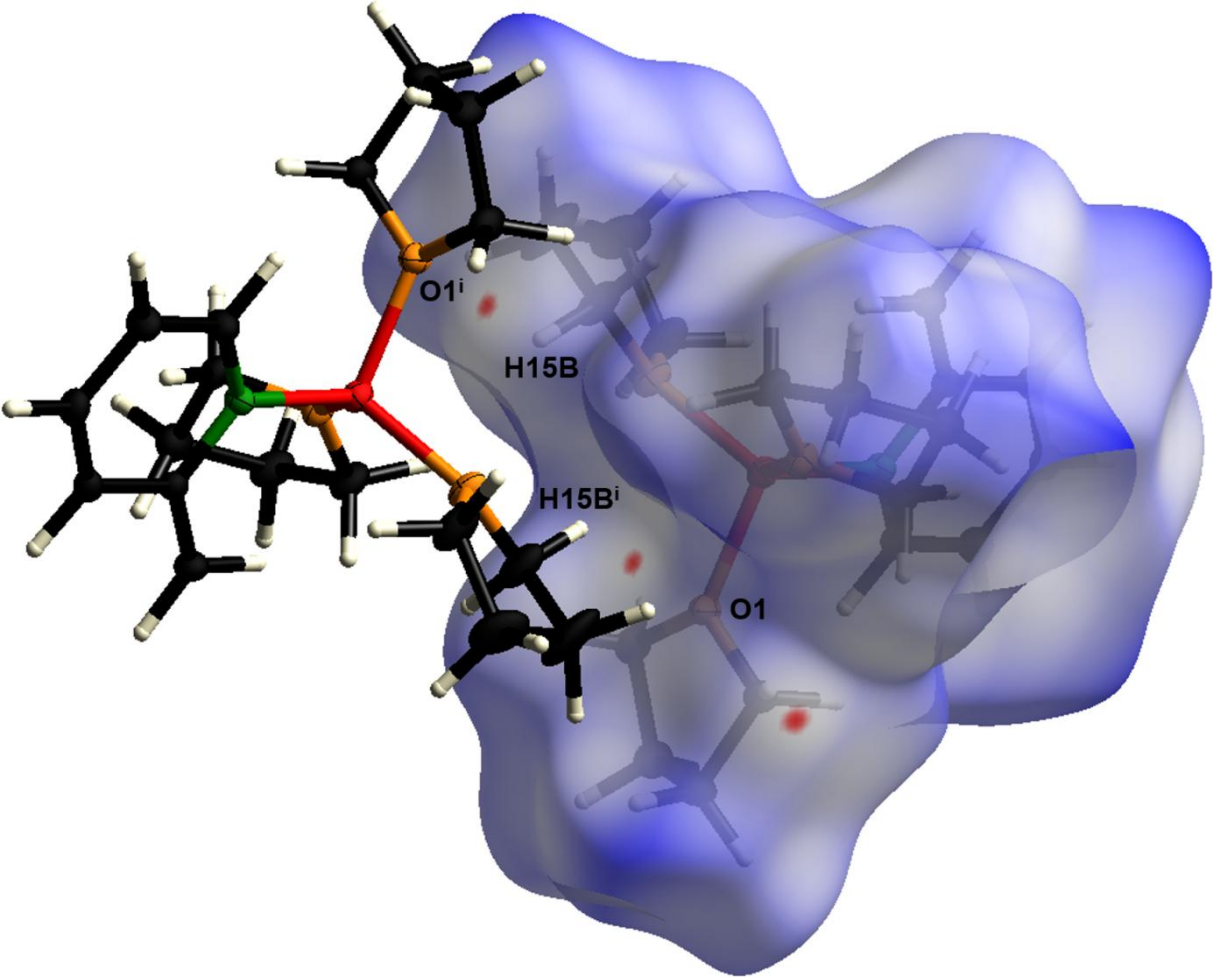
Hirshfeld surface analysis of **1** showing close contacts in the crystal. The weak hydrogen bond between O1^i^ and H15*B* is labeled. [Symmetry code: (i) 1 − *x*, 1 − *y*, 1 − *z*].

**Figure 7:**
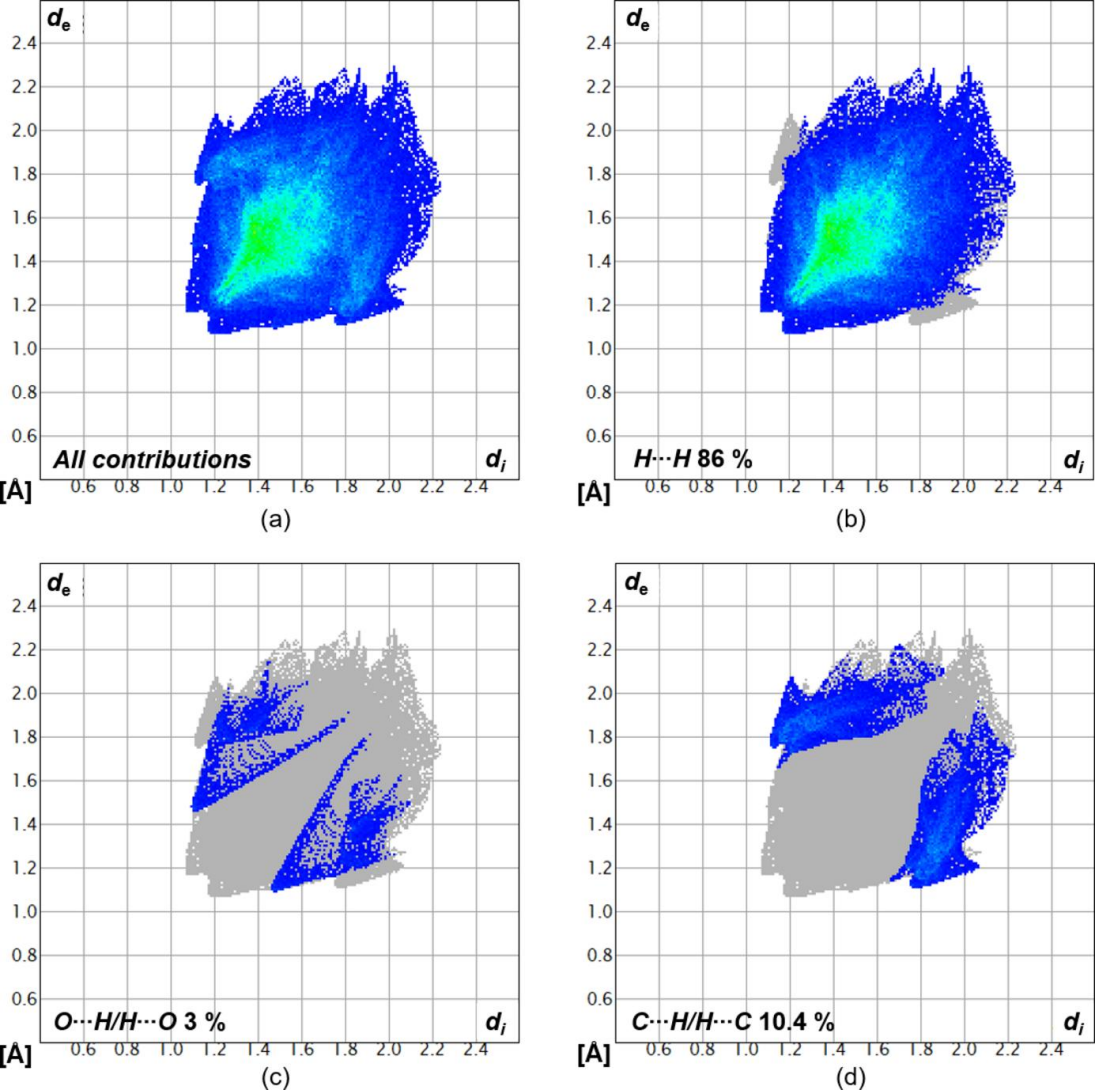
Two-dimensional fingerprint plots for compound 1, showing (*a*) all contributions and (*b*)–(*d*) contributions between specific inter­acting atom pairs (blue areas).

Fig. 6 ▸ shows the van der Waals inter­actions in the form of a Hirshfeld surface analysis mapped over *d*_norm_ in the range −0.02 to 1.61 a.u. (Spackman & Jayatilaka, 2009 ▸) generated by *CrystalExplorer21* (Spackman *et al.*, 2021 ▸) using red dots to represent close contacts. To visualize the percentages of the respective inter­actions, two-dimensional fingerprint plots (McKinnon *et al.*, 2007 ▸) were generated and are illustrated in Fig. 7 ▸. They show that inter­actions between H⋯H have the greatest influence (86%) to the packing of mol­ecules in the solid state. Inter­actions between O⋯H and C⋯H, as well as reciprocal contacts, contribute less to the crystal packing and can only be seen as spikes in the fingerprint plots with 3% and 10.4% contributions, respectively.

Due to its deprotonation, a partial negative charge at the CH_2_ substituent would be expected, but no distinct coordination points could be observed. The closest contact is C1⋯H13*B* at 2.97 Å but no specific inter­molecular inter­actions can be observed.

## 4. Database survey

A search of the Cambridge Crystallographic Database (WebCSD, November 2023; Groom *et al.*, 2016 ▸) for li­thia­ted 2-picoline or li­thia­ted 2-methyl­pyridine leads to the previously discussed structures **2** (Ott *et al.*, 2009 ▸), **2** and **5** (Kennedy *et al.*, 2014 ▸; Brouillet *et al.*, 2020 ▸). A few other li­thia­ted solid state structures of 2-picoline were published, for example bis­(μ2-dimesitylborinato)bis­(2-methyl­pyridine)­dilithium (ROLRIU; Saravana *et al.* (2009 ▸). However, it should be mentioned that the above structure and many other lithium 2-picoline complexes do not include the deprotonation of the methyl substituent and thus differ from the solid-state structures, accordingly this research. For example, bis­(μ_2_-tetra­hydro­borato)tetra­kis­(2-methyl­pyridine)­dilithium (HIWYOC; Gálvez Ruiz *et al.*, 2008 ▸). Compared to the few li­thia­ted structures of 2-picoline, there are many other coordination complexes with neutral 2-picoline. For example, between 2-picoline and transition metals, such as *trans*-di­iodo­bis­(2-picoline)platinum(II) (KARVEE; Tessier & Rochon, 1999 ▸) or between 2-picolyl cations and different anions, for example bis­(2-methyl­pyridinium)tetra­bromo­copper(II) (BACHOD; Luque *et al.*, 2001 ▸).

## 5. Synthesis and crystallization

On account of the air-sensitive nature of organolithium compounds, it was crucial to work with Schlenk techniques under an argon atmosphere. Pre-dried and distilled tetra­hydro­furan (1.00 ml) was added to an evacuated 25 ml Schlenk flask and 2-picoline (0.09 g, 1.00 mmol, 1.00 eq.) was added. After cooling down the reaction mixture to 193 K, *n*-butyl­lithium (2.5 *M* in hexane, 0.44 ml, 1.10 mmol, 1.10 eq.) was added. The resulting orange-colored suspension was heated up to 233 K over the period of 1 h. Afterwards the mixture was layered over by *n*-pentane (2.00 ml) and stored at 193 K. After 24 h, orange block-shaped crystals of the title compound were obtained.

## 6. Refinement

Crystal data, data collection and structure refinement details are summarized in Table 3 ▸. Hydrogen atoms except for H1*A* and H1*B* were positioned geometrically (C—H = 0.95–1.00 Å) and were refined using a riding model, with *U*_iso_(H) = 1.2*U*_eq_(C) for CH_2_ and CH hydrogen atoms and *U*_iso_(H) = 1.5*U*_eq_(C) for CH_3_ hydrogen atoms. The hydrogen atoms H1*A* and H1*B* were refined freely.

## Supplementary Material

Crystal structure: contains datablock(s) I. DOI: 10.1107/S2056989023010873/hb8083sup1.cif

Structure factors: contains datablock(s) I. DOI: 10.1107/S2056989023010873/hb8083Isup2.hkl

CCDC reference: 2320593

Additional supporting information:  crystallographic information; 3D view; checkCIF report

## Figures and Tables

**Table 1:** Selected geometric parameters (Å, °)

Li1—O1	1.9493 (16)	N1—C6	1.3479 (10)
Li1—O2	1.9698 (15)	C2—C3	1.4548 (10)
Li1—O3	1.9576 (15)	C3—C4	1.3664 (11)
Li1—N1	2.0131 (16)	C4—C5	1.4196 (12)
N1—C2	1.4017 (10)	C5—C6	1.3855 (11)
			
O1—Li1—O2	103.75 (7)	O2—Li1—N1	115.29 (7)
O1—Li1—O3	105.69 (7)	O3—Li1—O2	113.22 (7)
O1—Li1—N1	106.33 (7)	O3—Li1—N1	111.51 (7)

**Table 2:** Hydrogen-bond geometry (Å, °)

*D*—H⋯*A*	*D*—H	H⋯*A*	*D*⋯*A*	*D*—H⋯*A*
C15—H15*B*⋯O1^i^	0.99	2.63	3.3695 (14)	131

Symmetry code: (i) 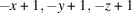 .

**Table 3:** Experimental details

Crystal data	
Chemical formula	[Li(C_6_H_6_N)(C_4_H_8_O)_3_]
*M*_r_	315.37
Crystal system, space group	Monoclinic, *P*2_1_/*n*
Temperature (K)	100
*a*, *b*, *c* (Å)	9.267 (3), 13.178 (4), 15.053 (5)
β (°)	94.437 (6)
*V* (Å^3^)	1832.7 (10)
*Z*	4
Radiation type	Mo *K*α
μ (mm^−1^)	0.08
Crystal size (mm)	0.39 × 0.29 × 0.21

Data collection	
Diffractometer	Bruker APEXII CCD
Absorption correction	Multi-scan (*SADABS*; Krause *et al.*, 2015 ▸)
*T*_min_, *T*_max_	0.493, 0.570
No. of measured, independent and observed [*I* > 2σ(*I*)] reflections	58129, 10326, 7724
*R*_int_	0.048
(sin θ/λ)_max_ (Å^−1^)	0.909

Refinement	
*R*[*F* ^2^ > 2σ(*F* ^2^)], *wR*(*F* ^2^), *S*	0.052, 0.159, 1.04
No. of reflections	10326
No. of parameters	226
H-atom treatment	H atoms treated by a mixture of independent and constrained refinement
Δρ_max_, Δρ_min_ (e Å^−3^)	0.68, −0.39

Computer programs: *APEX2* and *SAINT* V8.40B (Bruker, 2016 ▸), *SHELXT* (Sheldrick, 2015*a* ▸), *SHELXL2014/7* (Sheldrick, 2015*b* ▸) and *OLEX2* (Dolomanov *et al.*, 2009 ▸).

## supplementary crystallographic information

### Crystal data

**Table:**

[Li(C_6_H_6_N)(C_4_H_8_O)_3_]	*F*(000) = 688
*M**_r_* = 315.37	*D*_x_ = 1.143 Mg m^−^^3^
Monoclinic, *P*2_1_/*n*	Mo *K*α radiation, λ = 0.71073 Å
*a* = 9.267 (3) Å	Cell parameters from 812 reflections
*b* = 13.178 (4) Å	θ = 2.7–17.2°
*c* = 15.053 (5) Å	µ = 0.08 mm^−^^1^
β = 94.437 (6)°	*T* = 100 K
*V* = 1832.7 (10) Å^3^	Block, orange
*Z* = 4	0.39 × 0.29 × 0.21 mm

### Data collection

**Table:**

Bruker APEXII CCD diffractometer	7724 reflections with *I* > 2σ(*I*)
φ and ω scans	*R*_int_ = 0.048
Absorption correction: multi-scan (SADABS; Krause *et al.*, 2015)	θ_max_ = 40.2°, θ_min_ = 2.1°
*T*_min_ = 0.493, *T*_max_ = 0.570	*h* = −15→14
58129 measured reflections	*k* = −23→20
10326 independent reflections	*l* = −27→23

### Refinement

**Table:**

Refinement on *F*^2^	Primary atom site location: dual
Least-squares matrix: full	Hydrogen site location: mixed
*R*[*F*^2^ > 2σ(*F*^2^)] = 0.052	H atoms treated by a mixture of independent and constrained refinement
*wR*(*F*^2^) = 0.159	*w* = 1/[σ^2^(*F*_o_^2^) + (0.0816*P*)^2^ + 0.2483*P*] where *P* = (*F*_o_^2^ + 2*F*_c_^2^)/3
*S* = 1.04	(Δ/σ)_max_ = 0.002
10326 reflections	Δρ_max_ = 0.68 e Å^−^^3^
226 parameters	Δρ_min_ = −0.39 e Å^−^^3^
0 restraints	

### Special details

**Table:**

Geometry. All esds (except the esd in the dihedral angle between two l.s. planes) are estimated using the full covariance matrix. The cell esds are taken into account individually in the estimation of esds in distances, angles and torsion angles; correlations between esds in cell parameters are only used when they are defined by crystal symmetry. An approximate (isotropic) treatment of cell esds is used for estimating esds involving l.s. planes.

### Fractional atomic coordinates and isotropic or equivalent isotropic displacement parameters (Å^2^)

**Table:**

	*x*	*y*	*z*	*U*_iso_*/*U*_eq_	Occ. (<1)
O1	0.54688 (6)	0.40545 (4)	0.69990 (4)	0.02005 (10)	
O2	0.25473 (6)	0.37020 (4)	0.59195 (3)	0.01990 (10)	
O3	0.40664 (7)	0.59499 (4)	0.60466 (4)	0.02251 (11)	
N1	0.27503 (7)	0.50208 (5)	0.78389 (4)	0.01709 (10)	
C1	0.05286 (9)	0.55119 (6)	0.70141 (5)	0.02052 (13)	
H1A	0.0958 (14)	0.5399 (11)	0.6444 (9)	0.031 (3)*	
H1B	−0.0492 (14)	0.5759 (10)	0.6979 (8)	0.026 (3)*	
C2	0.13184 (8)	0.53679 (5)	0.78198 (4)	0.01576 (11)	
C3	0.07194 (8)	0.55663 (5)	0.86681 (5)	0.01810 (12)	
H3	−0.0262	0.5774	0.8673	0.022*	
C4	0.15372 (9)	0.54601 (6)	0.94577 (5)	0.02027 (13)	
H4	0.1128	0.5601	1.0004	0.024*	
C5	0.29996 (9)	0.51389 (6)	0.94596 (5)	0.02199 (14)	
H5	0.3600	0.5070	0.9997	0.026*	
C6	0.35063 (8)	0.49311 (6)	0.86368 (5)	0.01989 (13)	
H6	0.4479	0.4704	0.8635	0.024*	
C7	0.55077 (9)	0.30305 (6)	0.73460 (5)	0.02141 (13)	
H7A	0.5091	0.2547	0.6892	0.026*	
H7B	0.4956	0.2983	0.7882	0.026*	
C8	0.70987 (9)	0.28060 (6)	0.75781 (6)	0.02488 (15)	
H8A	0.7577	0.2567	0.7051	0.030*	
H8B	0.7231	0.2292	0.8057	0.030*	
C9	0.76766 (10)	0.38425 (7)	0.78926 (6)	0.02664 (16)	
H9A	0.7487	0.3970	0.8521	0.032*	
H9B	0.8729	0.3902	0.7828	0.032*	
C10	0.68127 (9)	0.45639 (6)	0.72633 (6)	0.02482 (15)	
H10A	0.6625	0.5210	0.7570	0.030*	
H10B	0.7351	0.4714	0.6736	0.030*	
C11	0.17241 (9)	0.29871 (6)	0.64195 (5)	0.02126 (13)	
H11A	0.1476	0.3291	0.6990	0.026*	
H11B	0.2288	0.2359	0.6549	0.026*	
C12	0.03647 (10)	0.27587 (7)	0.58261 (6)	0.02632 (16)	
H12A	−0.0410	0.3257	0.5917	0.032*	
H12B	0.0002	0.2066	0.5933	0.032*	
C13	0.09069 (11)	0.28551 (6)	0.48928 (6)	0.02723 (16)	
H13A	0.1429	0.2237	0.4726	0.033*	
H13B	0.0101	0.2991	0.4438	0.033*	
C14	0.19207 (10)	0.37589 (6)	0.50112 (5)	0.02495 (15)	
H14A	0.2684	0.3722	0.4587	0.030*	
H14B	0.1380	0.4402	0.4910	0.030*	
C15	0.37218 (13)	0.62291 (7)	0.51309 (5)	0.0318 (2)	
H15A	0.2719	0.6025	0.4934	0.038*	
H15B	0.4393	0.5896	0.4741	0.038*	
C16	0.3882 (2)	0.73688 (8)	0.50938 (7)	0.0516 (4)	
H16A	0.4885	0.7565	0.4988	0.062*	0.717 (5)
H16B	0.3209	0.7666	0.4621	0.062*	0.717 (5)
H16C	0.4604	0.7554	0.4670	0.062*	0.283 (5)
H16D	0.2945	0.7684	0.4889	0.062*	0.283 (5)
C17	0.3499 (2)	0.76969 (9)	0.60076 (10)	0.0299 (4)	0.717 (5)
H17A	0.3994	0.8339	0.6188	0.036*	0.717 (5)
H17B	0.2441	0.7792	0.6021	0.036*	0.717 (5)
C17A	0.4337 (5)	0.7718 (2)	0.59564 (19)	0.0232 (9)	0.283 (5)
H17C	0.3801	0.8339	0.6100	0.028*	0.283 (5)
H17D	0.5385	0.7874	0.6000	0.028*	0.283 (5)
C18	0.40128 (13)	0.68536 (6)	0.65944 (6)	0.0325 (2)	
H18A	0.4987	0.7009	0.6879	0.039*	0.717 (5)
H18B	0.3344	0.6749	0.7068	0.039*	0.717 (5)
H18C	0.4750	0.6825	0.7106	0.039*	0.283 (5)
H18D	0.3045	0.6938	0.6821	0.039*	0.283 (5)
Li1	0.36132 (16)	0.47086 (10)	0.66828 (9)	0.0189 (2)	

### Atomic displacement parameters (Å^2^)

**Table:**

	*U*^11^	*U*^22^	*U*^33^	*U*^12^	*U*^13^	*U*^23^
O1	0.0182 (2)	0.0180 (2)	0.0236 (2)	−0.00114 (17)	−0.00041 (18)	0.00186 (17)
O2	0.0242 (3)	0.0201 (2)	0.01507 (19)	−0.00422 (18)	−0.00091 (17)	−0.00015 (16)
O3	0.0354 (3)	0.0150 (2)	0.0172 (2)	−0.00083 (19)	0.0025 (2)	0.00046 (16)
N1	0.0175 (3)	0.0193 (2)	0.0143 (2)	0.00114 (19)	0.00059 (18)	−0.00134 (17)
C1	0.0204 (3)	0.0227 (3)	0.0180 (3)	0.0024 (2)	−0.0009 (2)	0.0007 (2)
C2	0.0176 (3)	0.0134 (2)	0.0163 (2)	−0.00083 (19)	0.0013 (2)	−0.00071 (18)
C3	0.0199 (3)	0.0164 (2)	0.0184 (3)	−0.0007 (2)	0.0041 (2)	−0.0020 (2)
C4	0.0243 (3)	0.0205 (3)	0.0164 (3)	−0.0030 (2)	0.0042 (2)	−0.0035 (2)
C5	0.0241 (3)	0.0269 (3)	0.0146 (2)	−0.0008 (3)	−0.0007 (2)	−0.0039 (2)
C6	0.0189 (3)	0.0244 (3)	0.0161 (3)	0.0010 (2)	−0.0005 (2)	−0.0029 (2)
C7	0.0211 (3)	0.0172 (3)	0.0257 (3)	−0.0014 (2)	−0.0002 (2)	0.0007 (2)
C8	0.0222 (3)	0.0237 (3)	0.0281 (4)	0.0028 (3)	−0.0017 (3)	0.0016 (3)
C9	0.0239 (4)	0.0303 (4)	0.0247 (3)	−0.0053 (3)	−0.0056 (3)	0.0020 (3)
C10	0.0244 (4)	0.0222 (3)	0.0271 (3)	−0.0064 (3)	−0.0027 (3)	0.0016 (3)
C11	0.0278 (4)	0.0185 (3)	0.0173 (3)	−0.0030 (2)	0.0007 (2)	0.0015 (2)
C12	0.0244 (4)	0.0244 (3)	0.0298 (4)	−0.0050 (3)	−0.0006 (3)	0.0003 (3)
C13	0.0362 (4)	0.0227 (3)	0.0213 (3)	−0.0047 (3)	−0.0076 (3)	−0.0016 (2)
C14	0.0351 (4)	0.0234 (3)	0.0156 (3)	−0.0047 (3)	−0.0025 (3)	0.0016 (2)
C15	0.0580 (6)	0.0211 (3)	0.0163 (3)	−0.0044 (3)	0.0028 (3)	0.0001 (2)
C16	0.1042 (11)	0.0230 (4)	0.0273 (4)	−0.0134 (5)	0.0030 (6)	0.0074 (3)
C17	0.0322 (10)	0.0159 (4)	0.0408 (7)	0.0010 (4)	−0.0016 (5)	−0.0044 (4)
C17A	0.028 (2)	0.0150 (9)	0.0251 (12)	−0.0041 (9)	−0.0038 (10)	0.0015 (8)
C18	0.0597 (6)	0.0175 (3)	0.0201 (3)	−0.0080 (3)	0.0022 (3)	−0.0030 (2)
Li1	0.0223 (6)	0.0184 (5)	0.0159 (5)	−0.0007 (5)	0.0011 (4)	−0.0001 (4)

### Geometric parameters (Å, º)

**Table:**

Li1—O1	1.9493 (16)	C10—H10A	0.9900
Li1—O2	1.9698 (15)	C10—H10B	0.9900
Li1—O3	1.9576 (15)	C11—H11A	0.9900
Li1—N1	2.0131 (16)	C11—H11B	0.9900
O1—C7	1.4465 (10)	C11—C12	1.5170 (12)
O1—C10	1.4433 (10)	C12—H12A	0.9900
O2—C11	1.4577 (10)	C12—H12B	0.9900
O2—C14	1.4454 (10)	C12—C13	1.5335 (14)
O3—C15	1.4384 (11)	C13—H13A	0.9900
O3—C18	1.4515 (10)	C13—H13B	0.9900
N1—C2	1.4017 (10)	C13—C14	1.5189 (12)
N1—C6	1.3479 (10)	C14—H14A	0.9900
C1—H1A	0.985 (14)	C14—H14B	0.9900
C1—H1B	0.998 (13)	C15—H15A	0.9900
C1—C2	1.3804 (10)	C15—H15B	0.9900
C2—C3	1.4548 (10)	C15—C16	1.5107 (14)
C3—H3	0.9500	C16—H16A	0.9900
C3—C4	1.3664 (11)	C16—H16B	0.9900
C4—H4	0.9500	C16—H16C	0.9900
C4—C5	1.4196 (12)	C16—H16D	0.9900
C5—H5	0.9500	C16—C17	1.510 (2)
C5—C6	1.3855 (11)	C16—C17A	1.411 (3)
C6—H6	0.9500	C17—H17A	0.9900
C7—H7A	0.9900	C17—H17B	0.9900
C7—H7B	0.9900	C17—C18	1.4755 (16)
C7—C8	1.5177 (12)	C17A—H17C	0.9900
C8—H8A	0.9900	C17A—H17D	0.9900
C8—H8B	0.9900	C17A—C18	1.535 (3)
C8—C9	1.5288 (13)	C18—H18A	0.9900
C9—H9A	0.9900	C18—H18B	0.9900
C9—H9B	0.9900	C18—H18C	0.9900
C9—C10	1.5248 (12)	C18—H18D	0.9900
			
C7—O1—Li1	119.84 (6)	H12A—C12—H12B	109.2
C10—O1—C7	109.73 (6)	C13—C12—H12A	111.4
C10—O1—Li1	125.99 (7)	C13—C12—H12B	111.4
C11—O2—Li1	113.13 (6)	C12—C13—H13A	111.5
C14—O2—C11	109.51 (6)	C12—C13—H13B	111.5
C14—O2—Li1	131.57 (6)	H13A—C13—H13B	109.3
C15—O3—C18	108.65 (6)	C14—C13—C12	101.59 (6)
C15—O3—Li1	129.97 (7)	C14—C13—H13A	111.5
C18—O3—Li1	112.93 (7)	C14—C13—H13B	111.5
C2—N1—Li1	119.19 (6)	O2—C14—C13	105.66 (6)
C6—N1—C2	118.22 (6)	O2—C14—H14A	110.6
C6—N1—Li1	122.58 (7)	O2—C14—H14B	110.6
H1A—C1—H1B	116.7 (11)	C13—C14—H14A	110.6
C2—C1—H1A	121.5 (8)	C13—C14—H14B	110.6
C2—C1—H1B	121.8 (7)	H14A—C14—H14B	108.7
N1—C2—C3	117.74 (6)	O3—C15—H15A	110.5
C1—C2—N1	119.98 (6)	O3—C15—H15B	110.5
C1—C2—C3	122.28 (7)	O3—C15—C16	105.94 (7)
C2—C3—H3	119.3	H15A—C15—H15B	108.7
C4—C3—C2	121.43 (7)	C16—C15—H15A	110.5
C4—C3—H3	119.3	C16—C15—H15B	110.5
C3—C4—H4	120.1	C15—C16—H16A	111.2
C3—C4—C5	119.83 (7)	C15—C16—H16B	111.2
C5—C4—H4	120.1	C15—C16—H16C	110.0
C4—C5—H5	121.7	C15—C16—H16D	110.0
C6—C5—C4	116.52 (7)	H16A—C16—H16B	109.1
C6—C5—H5	121.7	H16C—C16—H16D	108.4
N1—C6—C5	126.19 (7)	C17—C16—C15	102.72 (9)
N1—C6—H6	116.9	C17—C16—H16A	111.2
C5—C6—H6	116.9	C17—C16—H16B	111.2
O1—C7—H7A	110.7	C17A—C16—C15	108.27 (13)
O1—C7—H7B	110.7	C17A—C16—H16C	110.0
O1—C7—C8	105.16 (6)	C17A—C16—H16D	110.0
H7A—C7—H7B	108.8	C16—C17—H17A	111.0
C8—C7—H7A	110.7	C16—C17—H17B	111.0
C8—C7—H7B	110.7	H17A—C17—H17B	109.0
C7—C8—H8A	111.4	C18—C17—C16	104.00 (10)
C7—C8—H8B	111.4	C18—C17—H17A	111.0
C7—C8—C9	102.00 (7)	C18—C17—H17B	111.0
H8A—C8—H8B	109.2	C16—C17A—H17C	110.5
C9—C8—H8A	111.4	C16—C17A—H17D	110.5
C9—C8—H8B	111.4	C16—C17A—C18	105.94 (18)
C8—C9—H9A	111.3	H17C—C17A—H17D	108.7
C8—C9—H9B	111.3	C18—C17A—H17C	110.5
H9A—C9—H9B	109.2	C18—C17A—H17D	110.5
C10—C9—C8	102.13 (7)	O3—C18—C17	107.55 (8)
C10—C9—H9A	111.3	O3—C18—C17A	103.68 (13)
C10—C9—H9B	111.3	O3—C18—H18A	110.2
O1—C10—C9	106.36 (7)	O3—C18—H18B	110.2
O1—C10—H10A	110.5	O3—C18—H18C	111.0
O1—C10—H10B	110.5	O3—C18—H18D	111.0
C9—C10—H10A	110.5	C17—C18—H18A	110.2
C9—C10—H10B	110.5	C17—C18—H18B	110.2
H10A—C10—H10B	108.6	C17A—C18—H18C	111.0
O2—C11—H11A	110.6	C17A—C18—H18D	111.0
O2—C11—H11B	110.6	H18A—C18—H18B	108.5
O2—C11—C12	105.55 (6)	H18C—C18—H18D	109.0
H11A—C11—H11B	108.8	O1—Li1—O2	103.75 (7)
C12—C11—H11A	110.6	O1—Li1—O3	105.69 (7)
C12—C11—H11B	110.6	O1—Li1—N1	106.33 (7)
C11—C12—H12A	111.4	O2—Li1—N1	115.29 (7)
C11—C12—H12B	111.4	O3—Li1—O2	113.22 (7)
C11—C12—C13	101.94 (7)	O3—Li1—N1	111.51 (7)
			
O1—C7—C8—C9	34.54 (8)	C14—O2—C11—C12	−11.21 (8)
O2—C11—C12—C13	31.49 (8)	C15—O3—C18—C17	4.90 (13)
O3—C15—C16—C17	−30.31 (15)	C15—O3—C18—C17A	−26.28 (19)
O3—C15—C16—C17A	1.4 (2)	C15—C16—C17—C18	32.73 (16)
N1—C2—C3—C4	2.67 (10)	C15—C16—C17A—C18	−17.3 (3)
C1—C2—C3—C4	−177.20 (7)	C16—C17—C18—O3	−23.87 (15)
C2—N1—C6—C5	0.86 (12)	C16—C17A—C18—O3	26.7 (3)
C2—C3—C4—C5	−0.71 (11)	C18—O3—C15—C16	16.25 (13)
C3—C4—C5—C6	−1.14 (11)	Li1—O1—C7—C8	−175.91 (7)
C4—C5—C6—N1	1.12 (13)	Li1—O1—C10—C9	150.06 (8)
C6—N1—C2—C1	177.20 (7)	Li1—O2—C11—C12	145.25 (7)
C6—N1—C2—C3	−2.67 (9)	Li1—O2—C14—C13	−164.78 (8)
C7—O1—C10—C9	−5.96 (9)	Li1—O3—C15—C16	161.28 (10)
C7—C8—C9—C10	−37.15 (9)	Li1—O3—C18—C17	−146.62 (11)
C8—C9—C10—O1	27.22 (9)	Li1—O3—C18—C17A	−177.80 (17)
C10—O1—C7—C8	−18.19 (8)	Li1—N1—C2—C1	−1.37 (10)
C11—O2—C14—C13	−14.18 (9)	Li1—N1—C2—C3	178.76 (6)
C11—C12—C13—C14	−39.06 (8)	Li1—N1—C6—C5	179.37 (8)
C12—C13—C14—O2	33.23 (9)		

### Hydrogen-bond geometry (Å, º)

**Table:**

*D*—H···*A*	*D*—H	H···*A*	*D*···*A*	*D*—H···*A*
C15—H15*B*···O1^i^	0.99	2.63	3.3695 (14)	131

Symmetry code: (i) −*x*+1, −*y*+1, −*z*+1.
